# A Patient with Unexpected Golden Brown Tubular Staining

**DOI:** 10.34067/KID.0000000000000366

**Published:** 2024-04-25

**Authors:** Bhavna Bhasin-Chhabra, Maxwell Smith

**Affiliations:** 1 Division of Nephrology and Hypertension, Mayo Clinic Arizona, Scottsdale, Arizona; 2 Division of Anatomic Pathology, Mayo Clinic Arizona, Scottsdale, Arizona

**Keywords:** pigment, lipofuscin, hemosiderin, melanin, aging, geriatric nephrology, kidney biopsy

## Abstract

**Figure:**
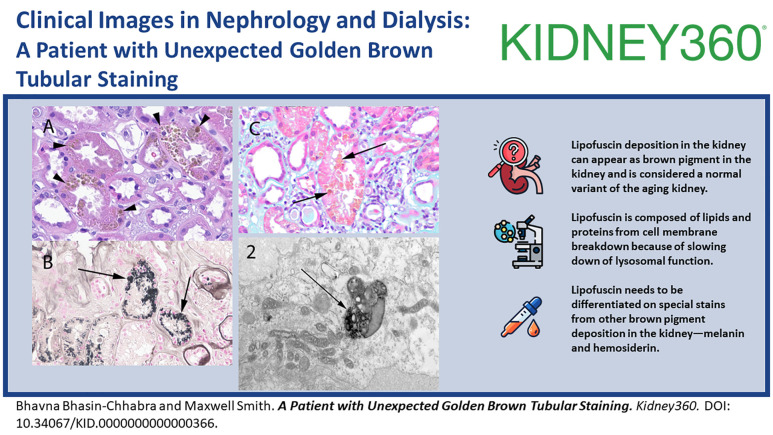

## Case Description

**Figure 1:**
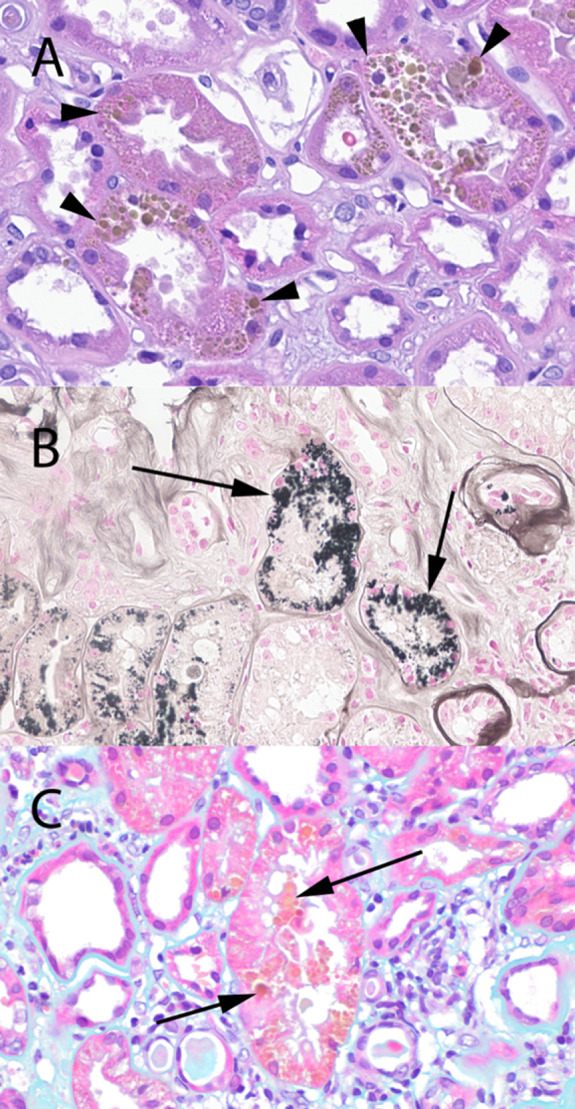
**Lipofuscin staining in the kidney**. (A) Numerous golden brown pigmented deposits of lipofuscin (arrowheads) in the cytoplasm of the tubular epithelial cells (H&E, 600×). (B) Positive (black staining, arrows) on Fontana-Masson stain (600×). (C) Deposits (arrow) are yellow to golden-brown in color without a dusty appearance on Papanicolaou stain (600×).

**Figure 2:**
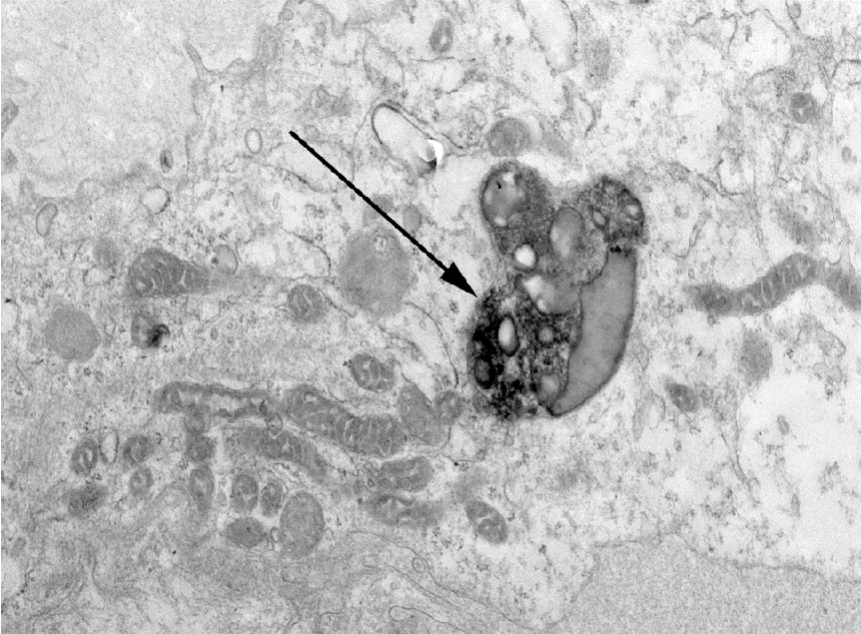
Characteristic cytoplasmic lipofuscin inclusion seen on transmission electron microscopy (arrow).

An 81-year-old man was seen in the nephrology clinic for evaluation of CKD stage 3B and was noted to have proteinuria of 3.7 g/g of creatinine on spot sample. Serum creatinine was 1.5–1.7 mg/dl at baseline. Urinalysis showed no blood, white blood cells, or red blood cells. Serological markers for membranous nephropathy (thrombospondin type 1 domain-containing 7A antibody and phospholipase A2 receptor antibody) were not detected. Serum and urine immunofixation studies were within normal range. His other comorbidities included hypertension, nephrolithiasis, mild cognitive impairment, and trigeminal neuralgia. Kidney biopsy was undertaken to evaluate the etiology of proteinuria and revealed hypertensive nephrosclerosis with changes of secondary focal segmental glomerular scarring as the likely etiology of the proteinuria. Incidental note was made of prominent golden brown granular deposits of variable size within the cytoplasm of the proximal tubular epithelial cells (Figure 1A). The biopsy was stained with hematoxylin and eosin, periodic acid–Shiff (PAS), trichrome, Jones silver, Prussian blue, Fontana-Masson (FM), Toluidine blue, and Papanicolaou stain. The deposits were golden brown to yellow color on hematoxylin and eosin and on unstained slides, weakly PAS positive, FM positive (Figure 1B), blue on Toluidine blue stain, and yellow to golden brown on Papanicolaou stain (Figure 1C). The deposits were negative for Prussian blue. Characteristic heterogeneous electron dense deposits were identified within the cytoplasm of the tubular epithelial cells (Figure 2). This constellation of histologic and electron microscopy findings support an interpretation of lipofuscin accumulation in the proximal tubules, a histologic finding associated with the normal aging process.

## Discussion

Lipofuscin deposition in the kidney is considered a normal physiological variant of aging in the kidney. Lipofuscin pigment is composed of proteins and lipids from cell membrane breakdown which may accumulate because of slowing down of lysosomal function or because of an altered state of lipid peroxidation. It has also been noted with vitamin E deficiency, high oxidative stress states (uremia and allograft rejection), and exposure to medications, such as amiodarone where lipofuscin deposition may be reversible. It can, however, be pathological in certain conditions, such as lysosomal storage and neurodegenerative diseases.^1^ It also has a pathological association with Hemansky-Pudlak syndrome where ceroid-like lipofuscin is associated with progressive renal injury and also other organ system involvement, including the lung and colon.^2^

Lipofuscin deposition in the kidney can lead to a blue or black appearance of the kidney which has been noted on gross examination of the kidney at the time of kidney donation.^3^ The differential diagnosis of brown pigment deposition in the kidney includes hemosiderin deposition and melanin deposition which has been noted in certain types of pigmented renal cell carcinoma and kidney involvement with metastatic melanoma.^4,5^ All these three pigments appear brown to golden brown when stained on hematoxylin and eosin staining. Lipofuscin stands out by its characteristic staining with lipid soluble agents, such as Sudan III and Oil Red O. Unfortunately, these staining modalities require fresh tissue that has yet to have the lipid dissolved out in processing. It is also weakly PAS positive, usually FM positive, blue by Toluidine blue, and golden yellow to light brown on Papanicolaou stain. The reactivity of lipofuscin for FM staining is a pitfall for melanin pigment which also stains positive with FM staining. However, melanin pigment has a more dusty appearance, is brown to black on Toluidine blue stain, and is brown to black on Papanicolaou stain.^6^ Prussian blue can help distinguish lipofuscin from hemosiderin deposition as lipofuscin is negative and hemosiderin is positive.

## Teaching Points

- Lipofuscin deposition in the kidney can appear as brown pigment in the kidney and is considered a normal variant of the aging kidney.
- Lipofuscin is composed of lipids and proteins from cell membrane breakdown because of slowing down of lysosomal function.
- Lipofuscin needs to be differentiated on special stains from other brown pigment deposition in the kidney—melanin and hemosiderin.
